# Synthesis of Novel 1T/2H-MoS_2_ from MoO_3_ Nanowires with Enhanced Photocatalytic Performance

**DOI:** 10.3390/nano10061124

**Published:** 2020-06-06

**Authors:** Wan Zhao, Xin Liu, Xiuru Yang, Chunxi Liu, Xiaoxiao Qian, Tao Sun, Wenya Chang, Jingjing Zhang, Zhi Chen

**Affiliations:** 1 College of Materials and Chemistry, China Jiliang University, 258 Xueyuan Street, Xiasha Higher Education Zone, Hangzhou 310018, China; 17826827277@163.com (W.Z.); yxr15957119921@126.com (X.Y.); 15867130507@163.com (C.L.); qxiao1116@126.com (X.Q.); 15958003262@163.com (T.S.); 18757556442@163.com (W.C.); jingbest@mail.ustc.edu.cn (J.Z.); 2 College of Standardization, China Jiliang University, 258 Xueyuan Street, Xiasha Higher Education Zone, Hangzhou 310018, China

**Keywords:** 1T/2H-MoS_2_ composite, molybdenum trioxide nanowires, photodegradation of antibiotic residue, hydrothermal method

## Abstract

Metallic 1T-phase MoS_2_ is a newly emerging and attractive catalyst since it has more available active sites and high carrier mobility in comparison with its widely used counterpart of semiconducting 2H-MoS_2_. Herein, 1T/2H-MoS_2_(N) (N: MoO_3_ nanowires were used to prepare 1T/2H-MoS_2_) was synthesized by using molybdenum trioxide (MoO_3_) nanowires as the starting material and applied in the photodegradation of antibiotic residue in water. Enhanced photocatalytic performance was observed on the obtained 1T/2H-MoS_2_(N), which was 2.8 and 1.3 times higher than those on 1T/2H-MoS_2_(P) (P: commercial MoO_3_ powder was used to prepare 1T/2H-MoS_2_) and 2H-MoS_2_, respectively. The active component responsible for the photodegradation was detected and a reaction mechanism is proposed.

## 1. Introduction

Environmental pollution, including water contamination, seriously threatens the survival and development of human beings [1]. Antibiotic residue is one of the refractory contaminants in water, and its efficient treatment is necessary for ecosystem protection [2,3]. Diverse techniques have emerged to realize their effective disposal [4,5,6]. Among these methods, photocatalysis has been intensively investigated, owing to its promising features, such as the direct conversion of solar power into chemical energy [7,8].

In recent decades, numerous photocatalysts such as TiO_2_ [9,10,11], ZnO [12], g-C_3_N_4_ [13,14], CdS [15], and WS_2_ [16] have been developed for the photocatalytic application. Among these photocatalysts, two-dimensional (2D) layered materials have attracted specific concern from academics and industry due to their prominent features, such as large specific surface area, strong electrical and thermal conductivity [17], high carrier mobility, and good transmittance [18,19]. The interaction between the different layers is the weak van der Waals force, which makes it possible to realize the exfoliation of the bulk 2D with improved properties [20,21].

Semiconducting molybdenum disulfide (2H-MoS_2_) is one of the promising 2D layered materials with excellent properties [22]. It can change from an indirect-bandgap semiconductor into a direct-bandgap one by reducing its number of layers [23,24]. Compared to the stable 2H-MoS_2_, metallic-phase MoS_2_ (1T-MoS_2_) is synthetic and metastable, and is easily converted into the stable 2H-MoS_2_. However, 1T-MoS_2_ has more active sites and higher electrical conductivity than 2H-MoS_2_, which makes it a promising candidate for widespread applications, including electrocatalysis [25], electrochemical cells [26], supercapacitors [27], and biosensors [28]. Nevertheless, the preparation of stable 1T-MoS_2_ is still a challenge. Previous studies found that 1T-MoS_2_ may coexist with 2H phase in monolayer. For instance, Wang et al. prepared 1T/2H-MoS_2_ through a hydrothermal method by using ammonium molybdate and thioacetamide as molybdenum and sulfur sources, whereby ammonium bicarbonate was added to provide intercalation ions and molecules for the formation of 1T-MoS_2_. The prepared 1T/2H-MoS_2_ had more active sites and showed improved electrochemical properties [29]. Ting et al. synthesized 1T/2H MoS_2_ through a microwave hydrothermal method using ammonium molybdate and thiourea as raw materials and applied it in the electrode material. Excellent capacitance and cyclic stability were observed at the 1T phase content of 73% [30]. Wang et al. annealed bulk 2H-MoS_2_ through a one-pot method under a mixture gas of Ar (argon) and phosphorous vapor to convert the 2H phase partially into a 1T phase. The obtained sample exhibited improved electrocatalytic properties [31]. Therefore, it can be seen that optimized performance can be realized in the presence of 1T-MoS_2_, which is influenced by the preparation method. Nevertheless, little attention was focused on the preparation of 1T-MoS_2_ by modulating the starting materials.

Herein, 1T/2H-MoS_2_(N) (N: MoO_3_ nanowires were used to prepare 1T/2H-MoS_2_) was successfully prepared by a facile hydrothermal method by using MoO_3_ nanowires as the starting material and applying visible-light-driven photocatalytic degradation of tetracycline hydrochloride in water. The as-synthesized 1T/2H-MoS_2_(N) shows enhanced photocatalytic activity, which is 2.8 and 1.3 times higher than those of 1T/2H-MoS_2_(P) (P: commercial MoO_3_ powder was used to prepare 1T/2H-MoS_2_), prepared from commercial MoO_3_ powder and 2H-MoS_2_, respectively. The prepared samples were analyzed and the active components for the photodegradation were studied. This article provides an alternative for the synthesis of 1T-MoS_2_-containing materials with high performance by modulating the starting materials.

## 2. Experiment

### 2.1. Materials

All of the reagents were used directly after purchased. Thioacetamide (C_2_H_5_NS, TAA), molybdenum trioxide powder (MoO_3_), ammonium molybdate tetrahydrate ((NH_4_)_6_Mo_7_O_24_∙4H_2_O) were purchased from Shanghai Aladdin Biochemical Technology Co. Ltd. (Shanghai, China). Urea (CH_4_N_2_O), tertiary butanol (TBA), ethylene diamine tetraacetic acid (EDTA) were obtained from Wuxi Prospect Chemical Reagent Co., Ltd. (Wuxi, China). Thiourea (CH_4_N_2_S), benzoquinone (BQ) were gotten from Shanghai Macklin Biochemical Co., Ltd. (Shanghai, China). Nitric acid (HNO_3_, 65%) was bought from Hangzhou Shuanglin Chemical reagent Co., Ltd. (Hangzhou, China). Tetracycline hydrochloride (TC-HCl) was obtained from Shanghai Jingchun Biochemical Technology Co., Ltd. (Shanghai, China). MoO_3_ nanowires (Figure S1) were prepared according to the previous report [32]. Typically, 1.0 g (NH_4_)_6_Mo_7_O_24_·4H_2_O was dissolved in 40.0 mL deionized water with magnetic stirring, and 6 mL of 65% HNO_3_ was added subsequently. After the complete dissolution, the solution was transferred into a Teflon-lined autoclave and kept at 180 °C for 12 h. The solid product was washed with deionized water until neutral and dried in vacuum at 60 °C.

### 2.2. Preparation

#### 2.2.1. Preparation of 1T/2H-MoS_2_(N)

1T/2H-MoS_2_(N) was synthesized by using the preformed MoO_3_ nanowire as the starting material; 1.0 g urea, 112.0 mg TAA, and 0.1 g synthesized MoO_3_ nanowires were dispersed in 80 mL deionized water and stirred for 2 h to form a uniform solution. Then, the solution was sealed in a Teflon-lined autoclave and heated at 200 °C for 12 h. After that, the synthesized 1T/2H-MoS_2_(N) was collected and washed with deionized water several times and dried by a freeze-drying process.2.2.2. Preparation of 2H-MoS_2_

2H-MoS_2_ was synthesized according to the previous report [33]. Typically, 4.57 g thiourea and 2.47 g ammonium molybdate tetrahydrate were dissolved in 70 mL deionized water and stirred for 2 h to form a uniform solution. Then, the solution was sealed in a Teflon-lined autoclave and heated at 180 °C for 24 h. The synthesized 2H-MoS_2_ was collected and washed with deionized water several times and dried by a freeze-drying operation.

#### 2.2.2. Preparation of 1T/2H-MoS_2_(P)

1T/2H-MoS_2_(P) was synthesized through an identical method as 1T/2H-MoS_2_(N), by replacing MoO_3_ nanowires with commercial MoO_3_ powder.

### 2.3. Characterization

SEM (scanning electron microscopy) images were acquired from the Hitachi SU8010 instrument (Hitachi, Tokyo, Japan). TEM (transmission electron microscopy) pictures were obtained from JEOL JEM-2100F equipment at 200 kV (JEOL, Tokyo, Japan). XRD (X-ray diffractometer) data were obtained from the DX-2700 and Bruker D2 Phaser using Cu kα radiation (Bruker, Karlsruhe, Germany). Raman spectra were obtained on a Renishaw inVia Raman spectrometer and the excitation wavelength was 514 nm (Renishaw, London, UK). UV–Vis DRS (ultraviolet–visible diffuse reflectance spectroscopy) was conducted on a Shimadzu UV-3600 ultraviolet-visible spectrophotometer (Shimadzu, Kyoto, Japan). XPS (X-ray photoelectron spectroscopy) was operated on an Axis Supra instrument (Shimadzu, Kyoto, Japan).

### 2.4. Photodegradation

The photocatalytic activities of all samples were evaluated by degrading TC-HCl solution under visible light (λ ≥ 420 nm), whereby a 300 W Xenon lamp served as the irradiation source. For this, 0.01 g prepared photocatalyst was immersed in a 100 mL TC-HCl solution (5 mg/L). The solution was kept constantly stirring for 1 h in the dark to reach the adsorption–desorption equilibrium. After that, the lamp was switched on, and a 3 mL solution was taken out every 10 min to analyze the concentration change, which was monitored at the maximum absorption of TC-HCl (356 nm) on the UV-2600 UV-Vis spectrophotometer (Sunny optical technology (group) Co., Ltd., Ningbo, China).

### 2.5. Trapping Experiment

Trapping experiments were carried out to explore the active species for photocatalysis. Different trapping agents such as TBA (2 mM), EDTA (0.5 mM), and BQ (2 mM) were added into the above solution for trapping hydroxyl radical (·OH), hole (h^+^), and superoxide radical (·O_2_^−^) respectively. The following steps were operated the same as those of photodegradation.

## 3. Result and Discussion

**Figure 1:**
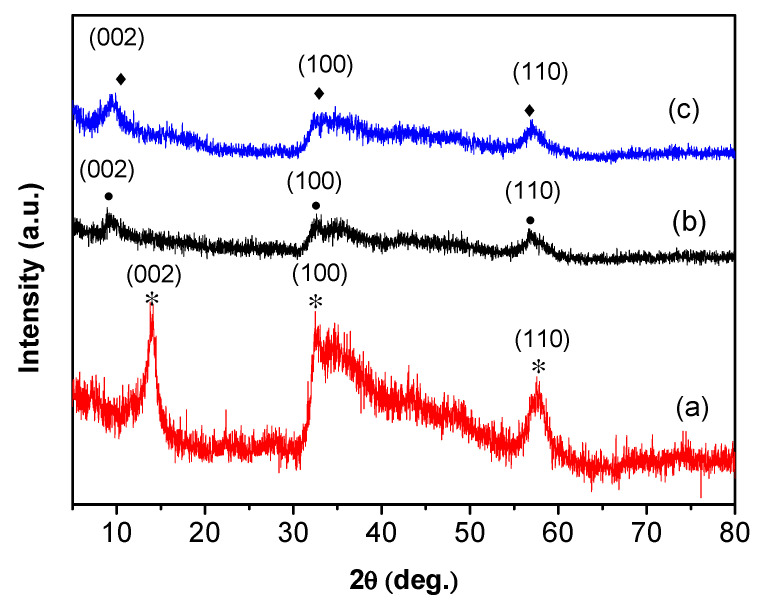
XRD patterns of (**a**) 2H-MoS_2_, (**b**) 1T/2H-MoS_2_(N), and (**c**) 1T/2H-MoS_2_(P).

XRD patterns of the prepared samples are shown in Figure 1. Curve (a) shows characteristic peaks at 2*θ* = 14.38°, 32.68°, and 57.76°, which correspond to (002), (100), and (110) of 2H-MoS_2_ (JCPDS No.37-1492), respectively. Curves (b) and (c) show that the (002) peak positions are at approximately 9.5°, which are both shifted to the left in comparison with curve (a). This may correspond to the characteristic peak of 1T-MoS_2_.

**Figure 2:**
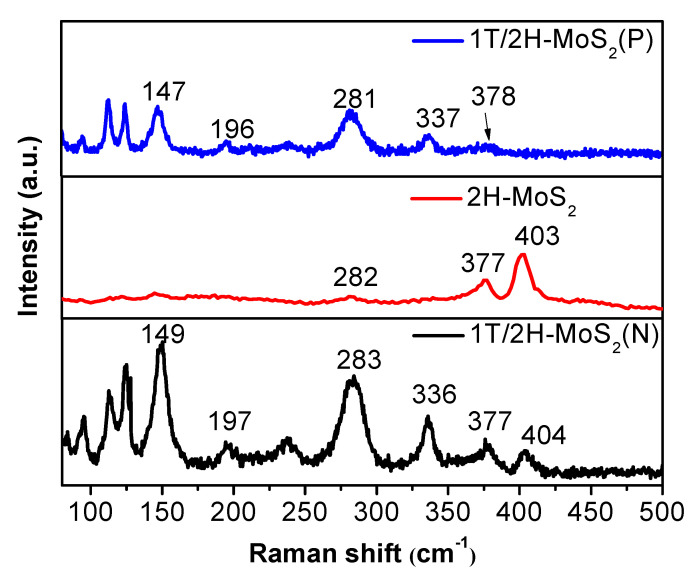
Raman spectra of 1T/2H-MoS_2_ (N), 2H-MoS_2_, and 1T/2H-MoS_2_(P).

Raman spectra were used to distinguish the 1T phase and 2H phase, shown in Figure 2. 1T/2H-MoS_2_(N) has six significant peaks located at 149, 197, 283, 336, 377, and 404 cm^−1^, respectively. The strong peaks at 149, 197, and 336 cm^−1^ could be assigned to the stretching vibration of the Mo–Mo and the phonon mode from 1T-MoS_2_, which confirms the presence of 1T-MoS_2_ [25]. The other three peaks at 284.7, 377, and 403 cm^−1^ may be attributed to the typical E_1g_, E^1^_2g_, and A_1g_ of 2H-MoS_2_, indicating the co-existence of 2H-MoS_2_. As for 2H-MoS_2_, only characteristic peaks at 283, 377, and 403 cm^−1^ were observed. The characteristic peaks of 1T/2H-MoS_2_(P) are similar to 1T/2H-MoS_2_(N); however, the prominent characteristic peaks at 378 and 404 cm^−1^ are absent due to the weak signals [29,34].

**Figure 3:**
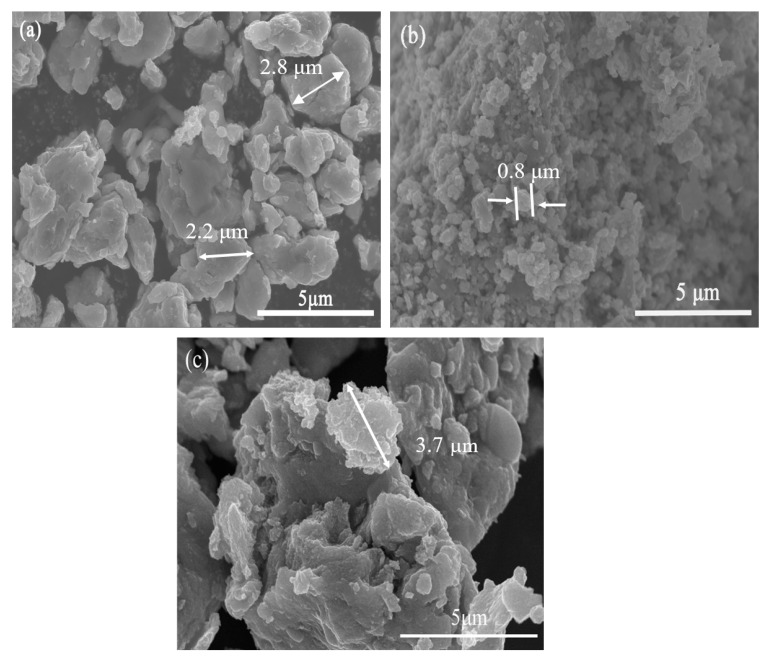
SEM images of (**a**) 1T/2H-MoS_2_(N), (**b**) 2H-MoS_2_, and (**c**) 1T/2H-MoS_2_(P).

SEM images of the prepared photocatalysts are shown in Figure 3. Figure 3a,c show that 1T/2H-MoS_2_(N) and 1T/2H-MoS_2_(P) are lamellar and stacked blocks with multiple layers. 1T/2H-MoS_2_(N) has particle sizes around 2.8 μm, which is obviously smaller than that of 1T/2H-MoS_2_(P) (3.7 μm). As shown in Figure 3b, the morphology of 2H-MoS_2_ is granular-like and the particle size is smaller (about 0.8 μm) with even distribution.

**Figure 4:**
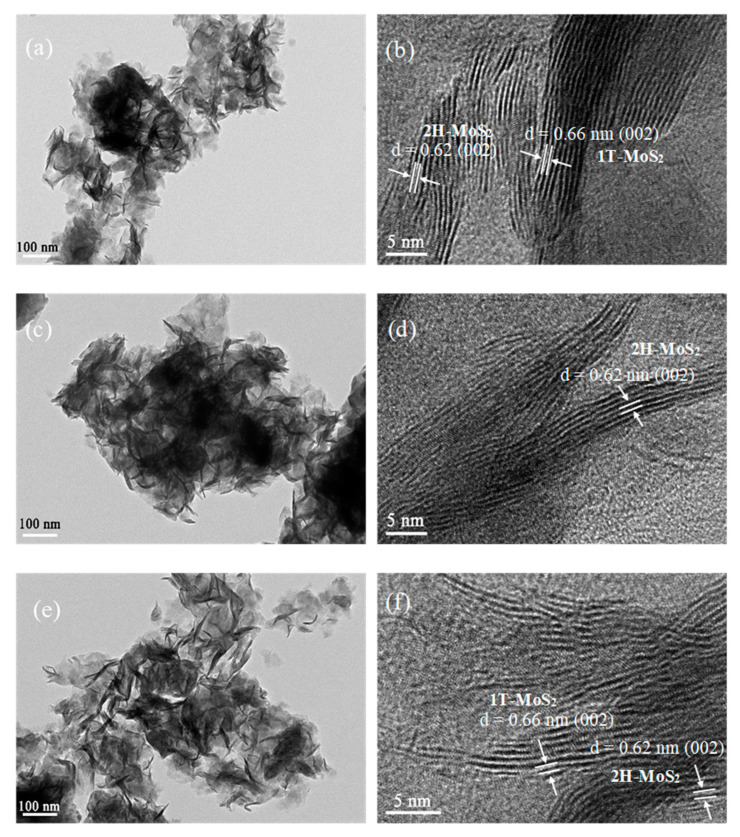
TEM images of: (**a**,**b**) 1T/2H-MoS_2_(N), (**c**,**d**) 2H-MoS_2,_ and (**e**,**f**) 1T/2H-MoS_2_(P); (**a**,**c**,**e**) low-magnification TEM images; (**b**,**d**,**f**) high-resolution TEM images.

TEM pictures of the prepared samples are given in Figure 4. The low-magnification pictures (Figure 4a,c,e) show that all of the prepared samples are flower-like and composed of the multilayered structure. The corresponding high-resolution pictures are shown in Figure 4b,d,f, and the spacing of the lattice fringe was measured and marked (Figures S2–S4). As shown in Figure 4b,f, the fringe spacing is around 0.66 nm, which corresponds to the (002) crystal plane of 1T-MoS_2_. This confirms the existence of 1T-MoS_2_ in both 1T/2H-MoS_2_(N) and 1T/2H-MoS_2_(P). In addition, the lattice spacing of 0.62 nm is observed on all three samples, which is assigned to the (002) crystal plane of 2H-MoS_2_. This confirms the presence of 2H-MoS_2_ in all of the samples. These results indicate the successful preparation of 1T/2H-MoS_2_ composites by using different starting materials.

**Figure 5:**
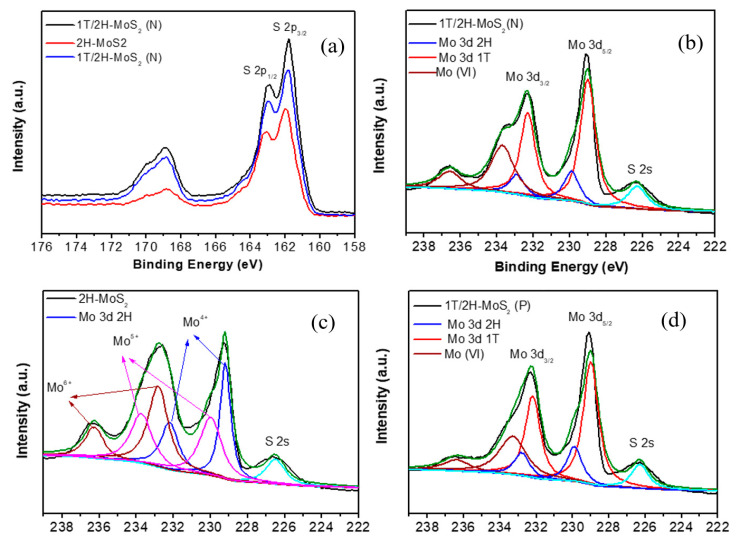
(**a**) S 2p XPS spectra of 1T/2H-MoS_2_(N), 2H-MoS_2_, and 1T/2H-MoS_2_(P); (**b**) Mo 3d XPS spectrum of 1T/2H-MoS_2_(N); (**c**) Mo 3d XPS spectrum of 2H-MoS_2_; (**d**) Mo 3d XPS spectrum of 1T/2H-MoS_2_(P).

X-ray photoelectron spectroscopy (XPS) was used to analyze the chemical composition. From Figure 5a, similar S spectra are observed for all three samples; however, the S 2p peaks of 2H-MoS_2_ shifted slightly to the higher energy direction compared with the other two samples. A distinct difference was observed on Mo 3d spectra, and therefore peak fitting was performed to further investigate the details of Mo 3d spectra. Mo peaks of 1T/2H-MoS_2_(N) are shown in Figure 5b, and two peaks at 229 and 232.3 eV may correspond to the 3d_5/__2_ and 3d_3/2_ of Mo^4+^ on 1T-MoS_2_. As for Mo 3d peaks assigned to the 2H-MoS_2_ (blue curves), these peaks are observed at 229.9 and 232.9 eV, respectively. They are shifted to a higher binding energy (about 1 eV) than those of 1T-MoS_2_, which is consistent with the previous report [34]. In addition, other peaks of Mo 3d are also observed, which can be attributed to the existence of Mo^6+^. As for 1T/2H-MoS_2_(P) in Figure 5d, similar situations were observed. The Mo 3d spectra of 2H-MoS_2_ are shown in Figure 5c; two peaks from Mo^4+^ are present at 229.2 and 232.2 eV, respectively. Additionally, the peaks from Mo^6+^ and Mo^5+^are also observed.

**Figure 6:**
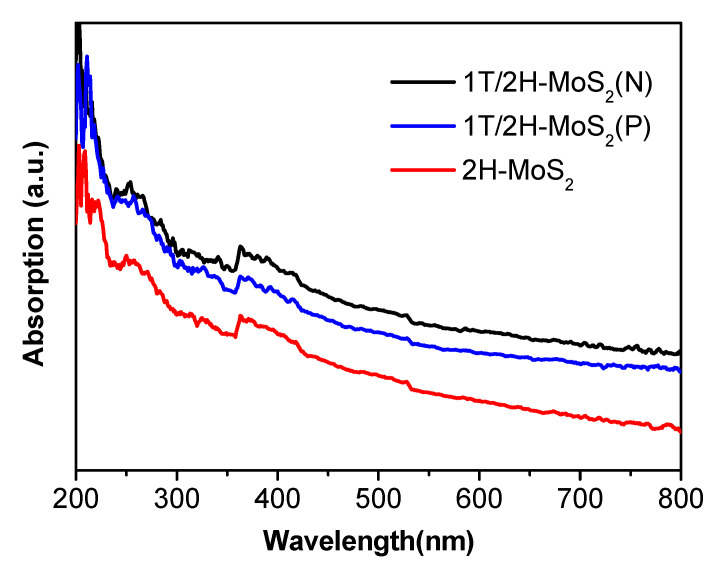
UV-Vis diffuse reflectance spectra of 1T/2H-MoS_2_(P), 2H-MoS_2_, and 1T/2H-MoS_2_(P).

UV-Vis diffuse reflectance spectra of the prepared samples are given in Figure 6. As shown, all three materials showed strong light absorption in the visible-light region. Increased absorption intensity was observed in 1T-MoS_2_ containing samples and the intensity sequence is 1T/2H-MoS_2_(N) > 1T/2H-MoS_2_(P) > 2H-MoS_2_.

**Figure 7:**
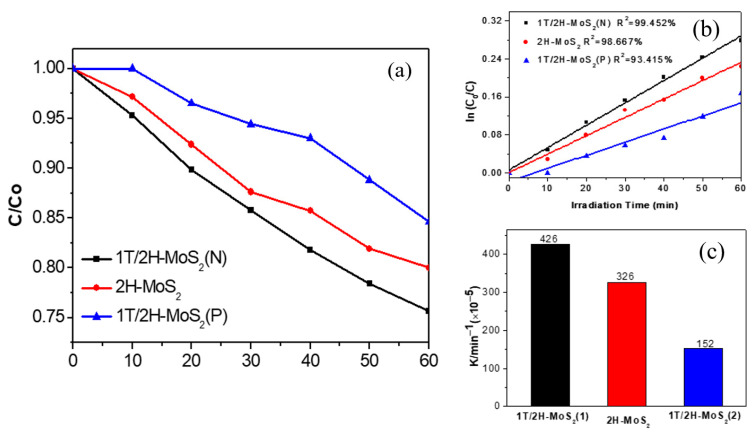
(**a**) Photodegradation of TC-HCl on 1T/2H-MoS_2_(N), 2H-MoS_2_, and 1T/2H-MoS_2_(P); (**b**) First-order kinetic fitting curve of TC-HCl degradation on 1T/2H-MoS_2_(N), 2H-MoS_2_, and 1T/2H-MoS_2_; (**c**) Histogram of the degradation reaction constant *k*.

The photocatalytic degradation of TC-HCl (5 mg/L) on the three prepared photocatalysts was carried out under visible light (λ ≥ 420 nm), as shown in Figure 7a. Results indicate that 25%, 20%, and 16% of TC-HCl were degraded at 60 min irradiation on 1T/2H-MoS_2_(N), 2H-MoS_2_, and 1T/2H-MoS_2_(P), respectively. First-order kinetic fitting was used to analyze the photocatalytic degradation, as shown in Figure 7b. The fitted curves are straight lines with high correlation coefficients (R^2^ > 93.4%), indicating that the photodegradation may obey first-order kinetics. The degradation reaction constant *k* was calculated according to the formula ln(*C_0_/C*) = *k*t, as illustrated in Figure 7c. The *k* values of 1T/2H-MoS_2_(N), 2H-MoS_2_, and 1T/2H-MoS_2_(P) were 4.26 × 10^−3^, 3.26 × 10^−3^, and 1.52 × 10^−3^ min^−1^, respectively. 1T/2H-MoS_2_(N) had the highest photodegradation efficiency, which was 1.3 and 2.8 times those of 2H-MoS_2_ and 1T/2H-MoS_2_(P), respectively.

**Figure 8:**
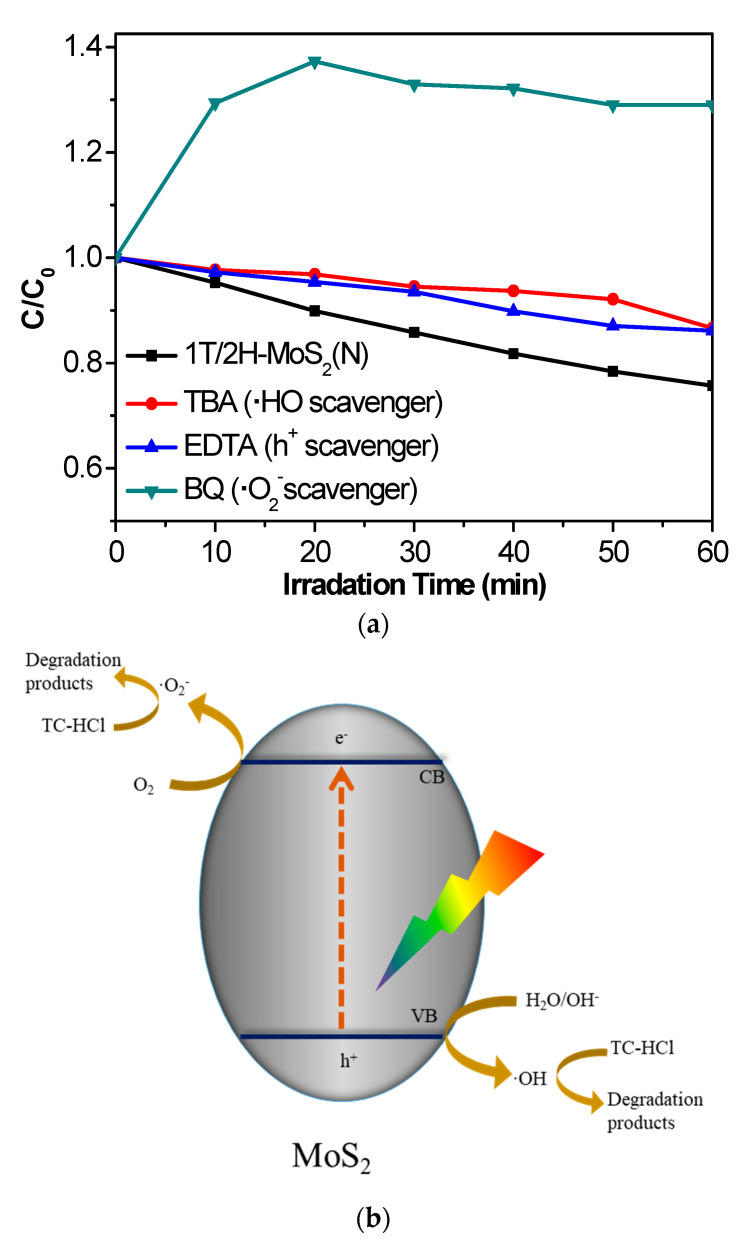
(**a**) Photocatalytic degradation of TC-HCl on 1T/2H-MoS_2_(N) without and with the addition of TBA, EDTA, or BQ. (**b**) Photodegradation diagram of 1T/2H-MoS_2_(N).

Trapping experiments were implemented to further analyze the active substances in the photocatalytic reactions on 1T/2H-MoS_2_(N), as shown in Figure 8a. Tert butyl alcohol (TBA, 2 mM) [35], benzoquinone (BQ, 0.5 mM) [36], and ethylene diamine tetraacetic acid (EDTA, 2 mM) [37] worked as the trapping agents for hydroxyl radical (·OH), superoxide radical (·O_2_^−^), and hole (h^+^), respectively, and were added into the TC-HCl solution. After the addition of trapping agents, all of the photocatalytic degradations of TC-HCl on 1T/2H-MoS_2_(N) were inhibited to some extent, indicating ·OH, ·O_2_^−^, and h^+^ are all responsible for the photocatalytic reaction. Figure 8b provides the photocatalytic mechanism diagram, illustrating the production process of three kinds of active particles. Electrons and holes may be produced under visible-light irradiation. The excited electrons react with O_2_ to form active ·O_2_^−^ species. The generated h^+^ can react with H_2_O to form active ·OH species. The formed ·OH, ·O_2_^−^, and h^+^ finally oxidize O_2_ into the target product.

## 4. Conclusions

1T/2H-MoS_2_(N) was successfully prepared by a simple hydrothermal process by using MoO_3_ nanowires as the starting material, which was applied to the photocatalytic degradation of TC-HCl. Enhanced photocatalytic performance was observed on 1T/2H-MoS_2_(N) in comparison with 2H-MoS_2_ and 1T/2H-MoS_2_(P), which indicates that the starting material should have an influence on the performance. The prepared samples were characterized and the active substances for photodegradation were investigated. Results demonstrate that the starting materials may have an influence on the performance of the product. This work provides an alternative to optimize the properties of given materials, which may benefit the design of high-performance catalysts for energy and environmental issues.

## Supplementary Materials

The following are available online at https://www.mdpi.com/2079-4991/10/6/1124/s1, Figure S1: SEM image of MoO_3_ nanowires, Figure S2: (a) Line scan of 2H-MoS_2_ image; (b) reduced FFT of 2H-MoS_2_; (c) Line scan of 1T-MoS_2_ image; (d) reduced FFT of 1T-MoS_2_, Figure S3: (a) Line scan of 2T-MoS_2_ image; (b) reduced FFT of 2T-MoS_2_, Figure S4: (a) Line scan of 2H-MoS_2_ image; (b) reduced FFT of 2H-MoS_2_; (c) Line scan of 1T-MoS_2_ image; (d) reduced FFT of 1T-MoS_2_.
