# Supplementary material for: Synthesis of Novel 1T/2H-MoS2 from MoO3 Nanowires with Enhanced Photocatalytic Performance
Source: Nanomaterials (Basel). 2020 Jun 6;10(6):1124. doi: 10.3390/nano10061124 (PMC7353267; doi:10.3390/nano10061124)
Supplement: Supplementary file 1 [file nanomaterials-10-01124-s001.pdf]

Supporting Information

Synthesis of Novel 1T/2H-MoS_2 from MoO_3 Nanowires with Enhanced Photocatalytic Performance

Wan Zhao [1], Xin Liu [2,*], Xiuru Yang [1], Chunxi Liu [1], Xiaoxiao Qian [1], Tao Sun [1], Wenya Chang [1], Jingjing Zhang [1] and Zhi Chen [1,*]

[1] College of Materials and Chemistry, China Jiliang University, 258 Xueyuan Street, Xiasha Higher Education Zone, Hangzhou 310018, China; 17826827277@163.com (W.Z.); yxr15957119921@126.com (X.Y.); 15867130507@163.com (C.L.); qxiao1116@126.com (X.Q.); 15958003262@163.com (T.S.); 18757556442@163.com (W.C.); jingbest@mail.ustc.edu.cn (J.Z.)

[2] College of Standardization, China Jiliang University, 258 Xueyuan Street, Xiasha Higher Education Zone, Hangzhou 310018, China

* Correspondence: liuxin@cjlu.edu.cn (X.L.); zchen@cjlu.edu.cn (Z.C.)

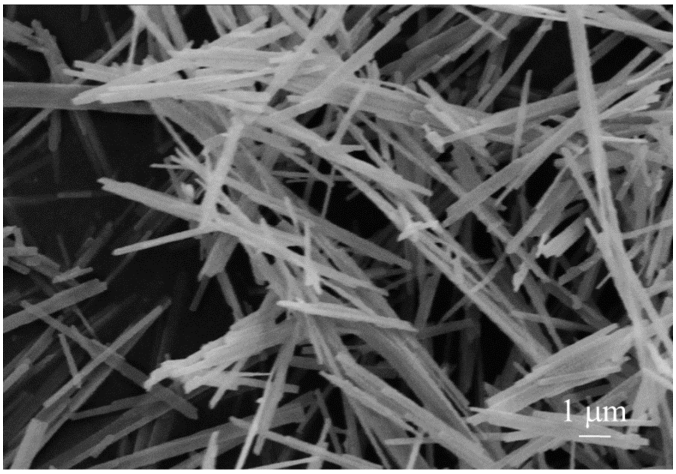

Fig. S1. SEM image of MoO_3 nanowires.

1T/2H-MoS_2 (N)

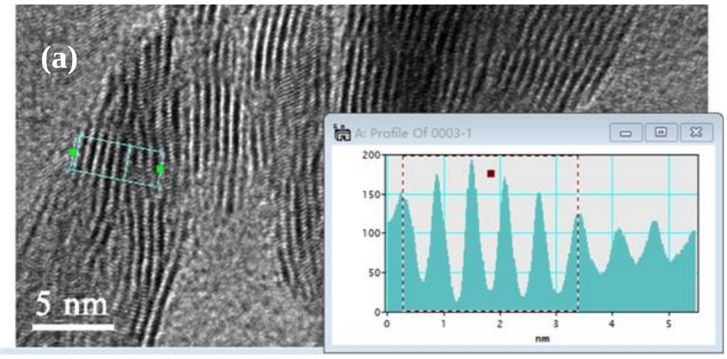

d=0.62 nm

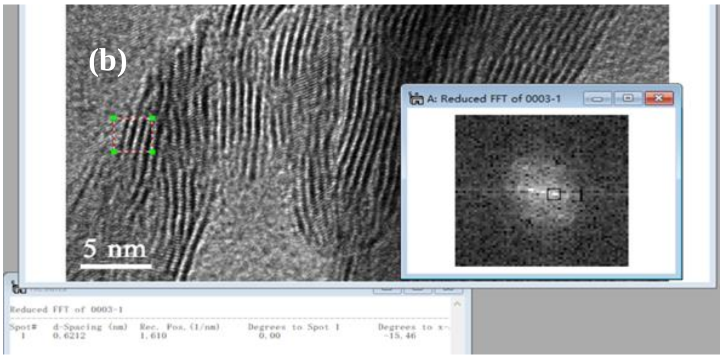

d=0.6212 n

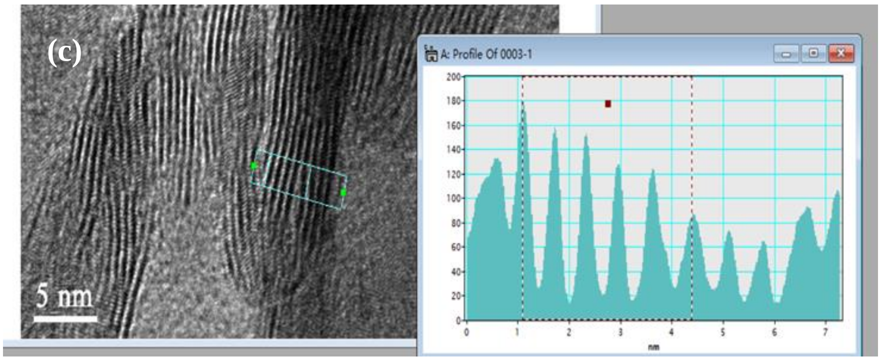

d=0.66 nm

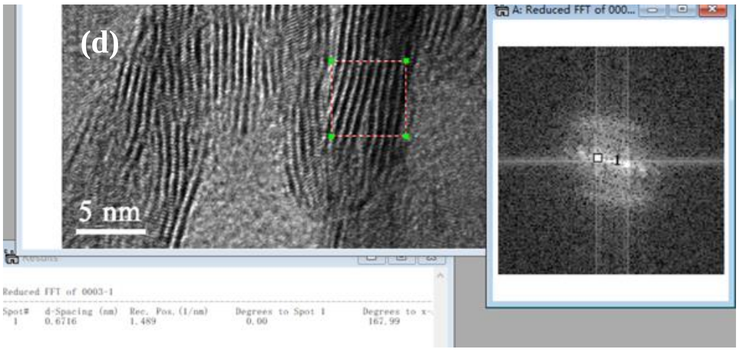

d=0.6716 nm

Fig. S2. (a) Line scan of 2H-MoS_2 image; (b) reduced FFT of 2H-MoS_2; (c) Line scan of 1T-MoS_2 image; (d) reduced FFT of 1T-MoS_2.

2H-MoS_2

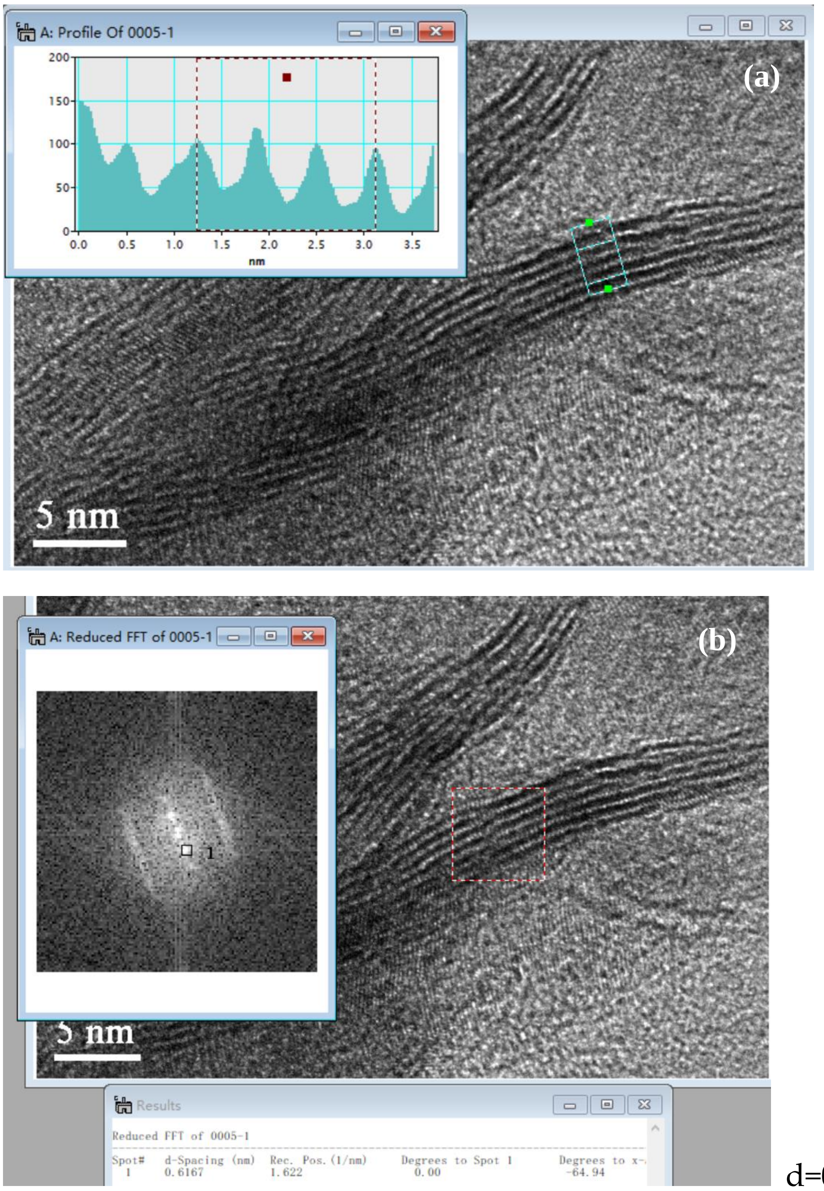

d=0.62 nm

d=0.6167 nm

Fig. S3. (a) Line scan of 2T-MoS_2 image; (b) reduced FFT of 2T-MoS_2.

1T/2H-MoS_2 (N)

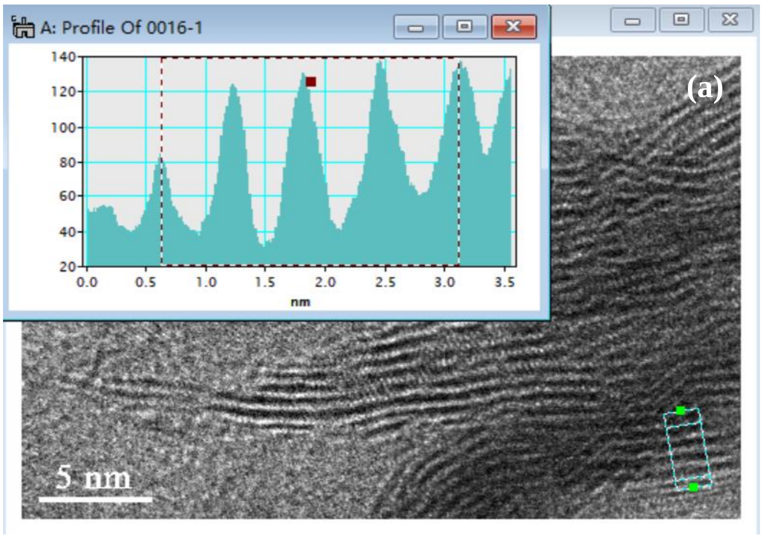

d=0.6218 nm

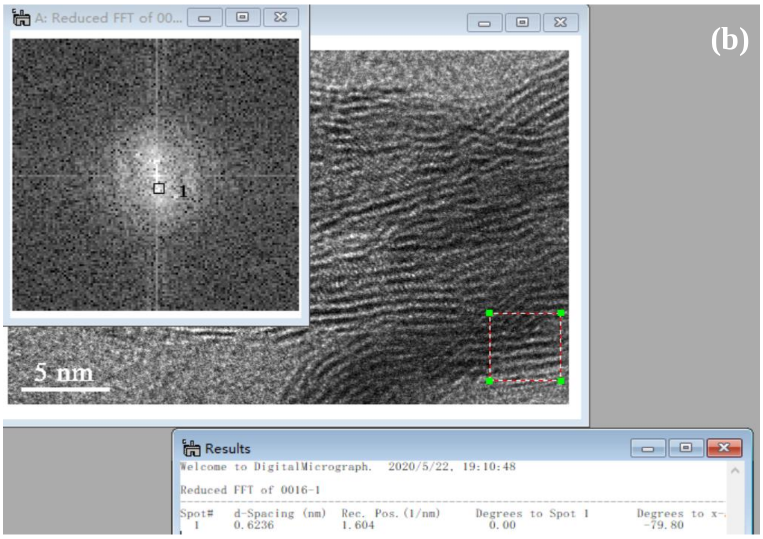

d=0.6236 nm

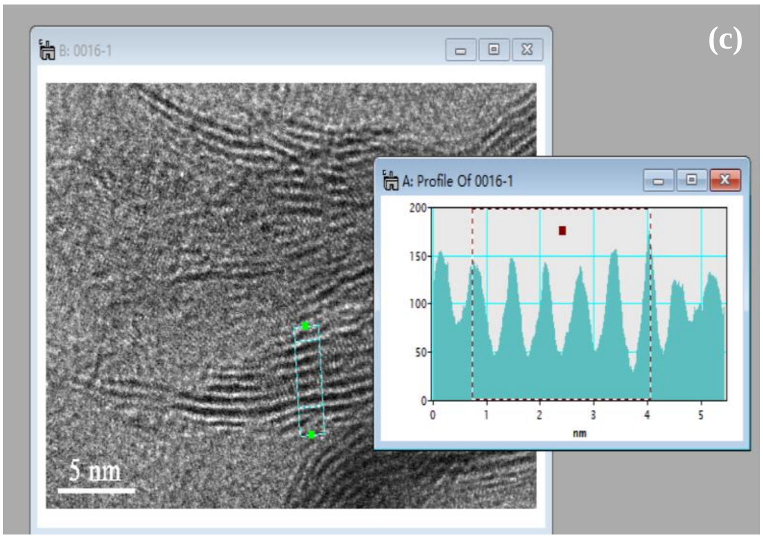

d=0.662 nm

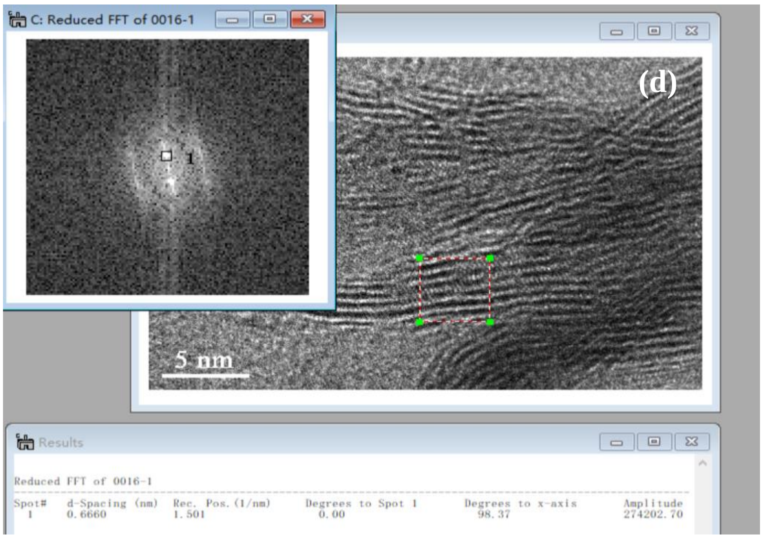

d=0.666 nm

Fig. S4. (a) Line scan of 2H-MoS_2 image; (b) reduced FFT of 2H-MoS_2; (c) Line scan of 1T-MoS_2 image; (d) reduced FFT of 1T-MoS_2.
